# Cardiac troponin and tropomyosin bind to F‐actin cooperatively, as revealed by fluorescence microscopy

**DOI:** 10.1002/2211-5463.12876

**Published:** 2020-06-18

**Authors:** Christopher Solís, John M. Robinson

**Affiliations:** ^1^ Department of Chemistry and Biochemistry South Dakota State University Brookings SD USA; ^2^ South Dakota State University Brookings SD USA; ^3^ Present address: Department of Physiology and Biophysics and Center for Cardiovascular Research College of Medicine University of Illinois at Chicago Chicago IL USA; ^4^ Present address: Wellzesta Health Research Institute Belmont NC USA

**Keywords:** binding isotherm, Monte Carlo, protein–protein interactions, quantitative imaging, stochastic simulation

## Abstract

In cardiac muscle, binding of troponin (Tn) and tropomyosin (Tpm) to filamentous (F)‐actin forms thin filaments capable of Ca^2+^‐dependent regulation of contraction. Tpm binds to F‐actin in a head‐to‐tail fashion, while Tn stabilizes these linkages. Valuable structural and functional information has come from biochemical, X‐ray, and electron microscopy data. However, the use of fluorescence microscopy to study thin filament assembly remains relatively underdeveloped. Here, triple fluorescent labeling of Tn, Tpm, and F‐actin allowed us to track thin filament assembly by fluorescence microscopy. It is shown here that Tn and Tpm molecules self‐organize on actin filaments and give rise to decorated and undecorated regions. Binding curves based on colocalization of Tn and Tpm on F‐actin exhibit cooperative binding with a dissociation constant *K*_d_ of ~ 0.5 µm that is independent of the Ca^2+^ concentration. Binding isotherms based on the intensity profile of fluorescently labeled Tn and Tpm on F‐actin show that binding of Tn is less cooperative relative to Tpm. Computational modeling of Tn‐Tpm binding to F‐actin suggests two equilibrium steps involving the binding of an initial Tn‐Tpm unit (nucleation) and subsequent recruitment of adjacent Tn‐Tpm units (elongation) that stabilize the assembly. The results presented here highlight the utility of employing fluorescence microscopy to study supramolecular protein assemblies.

## Graphical abstract

This work describes how a microscopy approach was used to quantify the assembly of thin filaments from troponin (Tn, green), tropomyosin (Tpm, red), and F‐actin (blue). The occupancy of the fluorescently labeled ligands Tn‐Tpm onto F‐actin filaments was quantified by two image analysis strategies: Tn‐Tpm colocalization and cross‐correlation analysis.

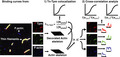

**Abbreviations**
Ac: actin
AF: Alexa Fluor
ddH_2_O: double distilled water
DTT: dithiothreitol
IANBD: N,N'‐dimethyl‐N‐(iodoacetyl)‐N'‐(7‐nitrobenz‐2‐oxa‐1,3‐diazol‐4‐yl) ethylenediamine
*K*_d_: dissociation constant
LB: labeling buffer
MOPS: 3‐(N‐morpholino)‐3‐propanesulfonic acid
*n*_H_: Hill coefficient
Tn: troponin
TnC: troponin C
TnI: troponin I
TnT: troponin T
Tpm: tropomyosin
WB: working buffer
*θ*: saturation constant

Myocardial contraction is regulated in thin filaments by troponin (Tn) and tropomyosin (Tpm) bound to F‐actin. Transient increases in cytosolic Ca^2+^ induce the rapid exchange of Mg^2+^ for Ca^2+^ in troponin C (TnC), which leads to conformational changes in Tn‐Tpm that enhance myosin binding and subsequent force development [1]. This fast activation depends on the linear integration of Tn and Tpm units throughout the actin filaments. The understanding of thin filament assembly, maintenance, and structure is an active research subject [2, 3, 4].

Assembly of Tn, Tpm, and F‐actin is sufficient to produce Ca^2+^‐regulated filaments. TnT N‐terminal domain bridges two neighboring Tpm units connected in a head‐to‐tail basis [5, 6, 7]. This arrangement has a high binding affinity for F‐actin (*K*_d_ ~ 0.5–2 μm) as a result of the concatenation of Tn‐Tpm units [8, 9]. It stands in contrast with the low binding affinity of single Tpm units binding to F‐actin (*K*_d_ ~ 400 μm) due to the weak noncovalent interactions between Tpm and F‐actin that support Tpm azimuthal diffusion. [10, 11]. Overall, this neighbor‐to‐neighbor Tn‐Tpm linkage decorates actin with two parallel chains that azimuthally diffuse in response to Ca^2+^ and myosin allosteric control [12].

Tn has a second low‐affinity actin binding site (*K*_d_ ~ 50 μm) located at the C‐terminal domain of TnI, which is responsible for locking the regulatory proteins in the blocked state of thin filaments as a function of the cytosolic Ca^2+^ concentration [3, 13]. With every heartbeat, cytoplasmic Ca^2+^ concentrations vary from < 100 nm to near 20 µm [14]. Ca^2+^ binding to the low‐affinity binding site of TnC retracts the TnI C‐terminal domain from the actin surface. This allows Tpm displacement by myosin due to strongly bound cross‐bridges [15, 16]. It has been proposed that the TnI C‐terminal binding of actin contributes significantly to the overall binding stability of Tn and Tpm to F‐actin [9, 17]. However, other reports have suggested that this interaction may not be significant [18, 19]. Since this TnI C‐terminal interaction with actin is sensitive to Ca^2+^, it is of interest to test the hypothesis that changing Ca^2+^ concentrations may affect the binding stability of Tn and Tpm to F‐actin.

Valuable knowledge about the mechanisms of supramolecular protein assembly has come from high‐resolution techniques such as superresolution and cryo‐electron microscopy [20, 21]. Although precise information can be obtained at nanoscopic scales, these techniques are time‐intensive and expensive for exhaustive data collection. Multichannel fluorescence microscopy is suitable for interrogating the assembly of proteins at mesoscopic scales when individual proteins are individually tagged with fluorophores [22].

Here, the assembly mechanism of thin filaments from Tn‐Tpm and F‐actin was studied using three‐color fluorescence microscopy. Tn‐Tpm decoration on actin filaments was quantified by colocalization and by cross‐correlation of the fluorescent intensity of decorated filaments. Results suggest that Tn and Tpm bind to F‐actin cooperatively, while Ca^2+^ does not play a significant role. However, Tn binding affinity is lower relative to Tpm. Stochastic simulations of Tn‐Tpm binding to F‐actin indicate that Tn‐Tpm assembly starts with the binding of a single Tn‐Tpm unit followed by incorporation of Tn‐Tpm units in a concatenated fashion.

## Materials and methods

### Protein expression and purification

Actin and Tpm were purified as described [23, 24] from bovine left ventricle acetone powder preparations. Recombinant single‐cysteine (C89) cardiac troponin C (TnC) from mouse, WT cardiac troponin I (TnI) from mouse, and WT cardiac TnT from adult rat plasmids were obtained from Herbert C. Cheung (University of Alabama at Birmingham) and expressed and purified as described [16, 25].

### Dye labeling of proteins

C89 in TnC was selectively labeled with AF 546 (Alexa Fluor 546, Life Technologies, Grand Island, NY, USA). Tpm was labeled at the single endogenous cysteine 190C with ATTO 655 (ATTO‐TEC, Siegen, Germany). Cysteines were reduced by dialysis against labeling buffer (LB: 3 m urea, 100 mm KCl, 1 mm EDTA, 50 mm MOPS (3‐(N‐morpholino)‐3‐propanesulfonic acid), pH 7.2) containing 5 mm DTT (dithiothreitol). Free DTT was removed by three dialysis steps against LB. Reduced proteins (100 μm) were labeled with a fivefold excess of dye molecule for 12 h at 4 °C under nitrogen with stirring. Labeling was terminated by the addition of 10 mm DTT. Unreacted dye molecules were removed by size‐exclusion chromatography (Sephacryl S‐100 HR, GE Life Sciences, Pittsburg, PA, USA) in LB using fast protein liquid chromatography (Akta GE Life Sciences). Fluorescent dye labeling was repeated until the labeling efficiency exceeded 95%. Protein concentration and labeling efficiencies were determined by absorption spectroscopy using the following extinction coefficients (m ^−1^·cm^−1^): Alexa 546, 104 000 at 555 nm; ATTO 655, 125 000 at 663 nm; Tn, 36 040 at 280 nm; Tpm, 23 230 at 280 nm; G‐actin, 48 300 at 280 nm. Typical labeling ratios for Tn and Tpm are > 0.95 and > 0.9, respectively. F‐actin was labeled with N,Nʹdimethyl‐N‐(iodoacetyl)‐N'‐(7‐nitrobenz‐2‐oxa‐1,3‐diazol‐4‐yl) ethylenediamine (IANBD) at C374 by combining F‐actin with fivefold excess of dye molecule as described previously [26]. Typical labeling efficiencies were 0.3 based on an extinction coefficient of 25 000 m ^−1^·cm^−1^ for IANBD.

### Preparation of the troponin complex

Fluorescently labeled cardiac Tn was reconstituted from TnC, TnI, and TnT using stepwise dialysis as described previously [25]. Briefly, TnC, TnI, and TnT were separately dialyzed in reconstitution buffer (50 mm Tris, 6 m urea, 500 mm NaCl, 5 mm CaCl_2_, 5 mm DTT, pH 8.0) and then mixed at a molar ratio of 1 : 1.2 : 1.4 TnC : TnI : TnT (10, 12, and 14 μm, respectively). The mixture was gently shaken for 2 h at room temperature and dialyzed against high salt buffer (1 m KCl, 20 mm MOPS, 1.25 mm MgCl_2_, 1.25 mm CaCl_2_, 1.5 mm DTT, pH 7.0). The urea was removed in four stepwise changes (from 6, 4, 2, and 0 m urea). KCl concentration was reduced by dialyzing against working buffer (WB: 75 mm KCl, 5 mm MgCl_2_, 2 mm EGTA, 5 mm 2‐mercaptoethanol, 50 mm MOPS) supplemented with KCl (from 1, 0.7, 0.5, 0.3, 0.15 m KCl). Uncomplexed TnI and TnT, which precipitate in WB, were removed by centrifugation at 10 000 ***g*** for 1 min. Reconstituted Tn was stored at −80 °C.

### Preparation of thin filaments

Thin filaments were reconstituted as described [16, 25]. Tn, Tpm, and F‐actin were mixed at a 1 : 1 : 7 stoichiometry (1 μm Tn, 1 μm Tpm, and 7 μm F‐actin) in WB for an hour at 4 °C. After 1‐h incubation, phalloidin AF 488 (Alexa Fluor 488, Life Technologies) was incorporated into thin filaments at 1 : 20 molar ratio of phalloidin: actin monomer (e.g., 0.35 μm phalloidin AF488 to 1 μm of thin filaments) and incubated for 20 min. Thin filament solutions at constant Tn: Tpm: F‐actin stoichiometry were prepared at 12.5, 25, 50, 100, 250, 500, and 750 nm, and the free Tn concentration was determined. For imaging purposes, filaments were diluted to 2 nm (2 nm Tn, 2 nm Tpm, and 14 nm F‐actin) and surface‐deposited on aminosilanized coverslips. The dilution and surface deposition step takes < 30 s on average, while imaging a single condition takes < 15 min. At these time intervals, dissociation of Tn‐Tpm from F‐actin is considered negligible as seen by the time‐dependent loss of Tn and Tpm localization on F‐actin (Fig. S1).

### Preparation of aminosilanized coverslips

Coverslips were aminosilanized as described previously [27]. Briefly, circular coverslips (No. 1, 25 mm, Fisher Scientific) were cleaned by successive sonication (10 min) in (a) 1% NaPO_4_, 1% SDS, 1% NaHCO_3_; (b) ddH_2_O (double distilled water); (c) acetone; and (d) 1 m NaOH. Cleaned coverslips were rinsed with ddH_2_O, dehydrated by immersion in methanol, and aminosilanized by incubation (2 periods of 10 min, interrupted by 1 min of sonication) with 1% (v/v) Vectabond (Vector Labs, Burlingame, CA, USA) and 5% (v/v) glacial acetic acid in methanol. The coverslips were extensively rinsed with methanol and then with ddH_2_O before drying in a dust‐free chamber. The aminosilanized coverslips were stored at room temperature in a closed container for up to 2 weeks.

### Microscopy and image analysis

Wide‐field epifluorescence images were acquired using an inverted IX71 Olympus microscope with TE‐cooled interline CCD camera (Clara, Andor, Belfast, Northern Ireland) using a 100× (N.A. 1.4) oil immersion objective with a resolution of 15 pixels·μm^−1^ (UPlanSApo, Olympus, Waltham, MA, USA). Excitation was from a Xe lamp (X‐Cite 120PC, Lumen Dynamics, Waltham, MA, USA). Filters (excitation: dichroic: emission) were FF01‐475/35: FF495: FF01‐550/88 (Semrock, Rochester, NY, USA) for AF 488 and IANBD, ET545/25: T565lpxr: ET605/70m (Chroma) for AF 546; and ET620/60: T660lpxr: ET700/75m (Chroma, Bellows Falls, VT, USA) for ATTO 655. Imaging length was calibrated using a dual‐axis linear scale (Edmund Industrial Optics, Barrington, NJ, USA). Measurements were performed at room temperature (18 ± 2 °C). Image processing and computer vision were performed using matlab (R2011b; MathWorks, Natick, MA, USA) and imagej 1.47t (National Institutes of Health, Bethesda, MD, USA).

Quantifying Tn‐Tpm bound to actin filaments provides a direct measure of the degree of Tn‐Tpm saturation across the F‐actin linear dimension. The saturation state of Tn‐Tpm (θ_Tn‐Tpm_) in thin filaments was determined by direct imaging of the fluorescence signal from Tn‐AF546, Tpm‐ATTO655, and F‐actin‐phalloidin‐AF488. The image processing protocol employs a binarization (Sobel edge detection) and distinction of decorated from undecorated regions in the actin filaments (see Fig. S2 for an extended description). A decorated region was identified from the combined fluorescence signal of Tn and Tpm. *θ*_Tn‐Tpm_ corresponds to the ratio between the number of pixels found in decorated regions and the number of pixels found in actin filaments, which is defined by Eqn (1):

(1) $$\theta_{\text{Tn}-\text{Tpm}}=\frac{\sum ∥x_{\text{Tn}-\text{Tpm}}\left(i\right)∥}{\sum ∥x_{\text{Actin}}\left(i\right)∥},$$

where the vectors ***x***_Tn‐Tpm_ and ***x***_Ac_ contain the recovered coordinates of F‐actin and Tn‐Tpm in a filament *i*, $∥\cdot ∥$ is the length operator for a subset *i*, and *θ*_Tn‐Tpm_ is the average saturation from *N* sampled filaments. Data derived from free Tn‐Tpm and F‐actin titrations were fit to the Hill equation fit to (Eqn 2):

(2) $$\theta_{\text{Tn}-\text{Tpm}}=\frac{{\left[\text{Tn}\right]}^{n_{H}}}{K_{d}+{\left[\text{Tn}\right]}^{n_{H}}},$$

where the degree of saturation of Tn‐Tpm on F‐actin, *θ*_TnTm_, depends on the total concentration of regulatory units [Tn‐Tpm]_0_ and defines the apparent dissociation constant *K*_d_ and the Hill coefficient *n*_H_. Cross‐correlation analysis of fluorescence intensities of Tn, ***I***(***x*** *_T_*_n_), and F‐actin, ***I***(***x***_Ac_), as a function of a filament coordinate were determined according to Eq (3):

(3) $$I\left(x_{\text{Tn}}\right)*I\left(x_{\text{Ac}}\right)=\sum_{i=0}^{N-l-1}\frac{E\left[\left(I\left(x_{\text{Tn}}\right)-〈I\left(x_{\text{Tn}}\right)〉\right)\cdot \left(I\left(x_{\text{Ac}}\right)-〈I\left(x_{\text{Ac}}\right)〉\right)\right]}{\sigma_{\text{Tn}}\cdot \sigma_{\text{Ac}}},$$

where ***I***(***x***_Tn_) and ***I***(***x***_Ac_) represent the fluorescent intensities in Tn and F‐actin using the filament positions ***x*** (derived from the F‐actin coordinates); σ_Tn_ and σ_Ac_ represent the standard deviations of the Tn and F‐actin intensities; and *l* and *N* correspond to the lag interval and the maximum extension of the inspection, respectively. The product of the cross‐correlation at *l* = 0 (i.e., where the correlation is expected to be maximal) is selected as the correlation value of a single filament. When comparing Tpm and F‐actin signals, Tn data are substituted for Tpm.

### Modeling and simulation

The stochastic simulation was modeled by the Monte Carlo approach using MATLAB (R2011b; MathWorks). Tn‐Tpm was considered to be the ligand, and F‐actin (Ac) a one‐dimensional lattice, where each binding space represents one regulatory unit. The reaction follows the scheme.

(4) $$\text{Tn}-\text{Tpm}+{\text{Ac}}_{7}\overset{k_{1}}{\underset{k_{-1}}{⇌}}\text{Tn}-\text{Tpm}-{\text{Ac}}_{7},$$

where *k*_1 _and *k*_−1 _define the forward and backward stochastic reaction constants, respectively [28]. Since the goal was to adopt a simple model, two assumptions were considered: (a) *k*_1_ is the diffusion‐controlled and activation‐controlled rate constant; (b) this assumption is equivalent for a ligand binding to an empty site, adjacent to one or two ligands already bound to the lattice. Positive cooperativity is considered in this model by introducing a coefficient that depends on the number of nearest‐neighbor ligands bound to the lattice. This coefficient affects the backward reaction constant *k*_−1_. For a given time interval d*t*, the deterministic solution of the rate of saturation of a linear lattice is given by

(5) $$\frac{d\theta }{dt}=k_{1}{\left[\text{Tn}-\text{Tpm}\right]}_{t}\left(\theta_{t}-1\right)\frac{{\left[\text{Ac}\right]}_{0}}{7}-k_{1}{\left(\frac{1}{\sigma }\right)}^{{〈n〉}_{t}}\theta_{t}\frac{{\left[\text{Ac}\right]}_{0}}{7},$$

where [Tn‐Tm]*_t_* is the free Tn‐Tpm concentration found at time *t*, *θ_t_* is the fractional saturation density, [Ac]_0_ is the total Ac concentration, *σ* is a cooperativity parameter, and $〈n〉$ is the average number of nearest‐neighbor interactions in a bound Tn‐Tpm unit at the previous iteration step *k*_−1_ (*n* = 0, 1, 2). Since the stochastic reaction constants were not known a priori, the *k*_1_/*k_−_*_1_ ratio was adjusted iteratively to match the output *K*_d_ from the simulation to the experimental dissociation constant *K*_d_.

### Data analysis

Data analysis was conducted in MATLAB (R2011b; MathWorks). Significance tests between pCa 9 and pCa 3 groups were determined by two‐tailed Student’s *t*‐tests with a significance level α = 0.05 of rejecting the null hypothesis.

## Results

### Localization of Tn‐Tpm on F‐actin

**Fig. 1:**
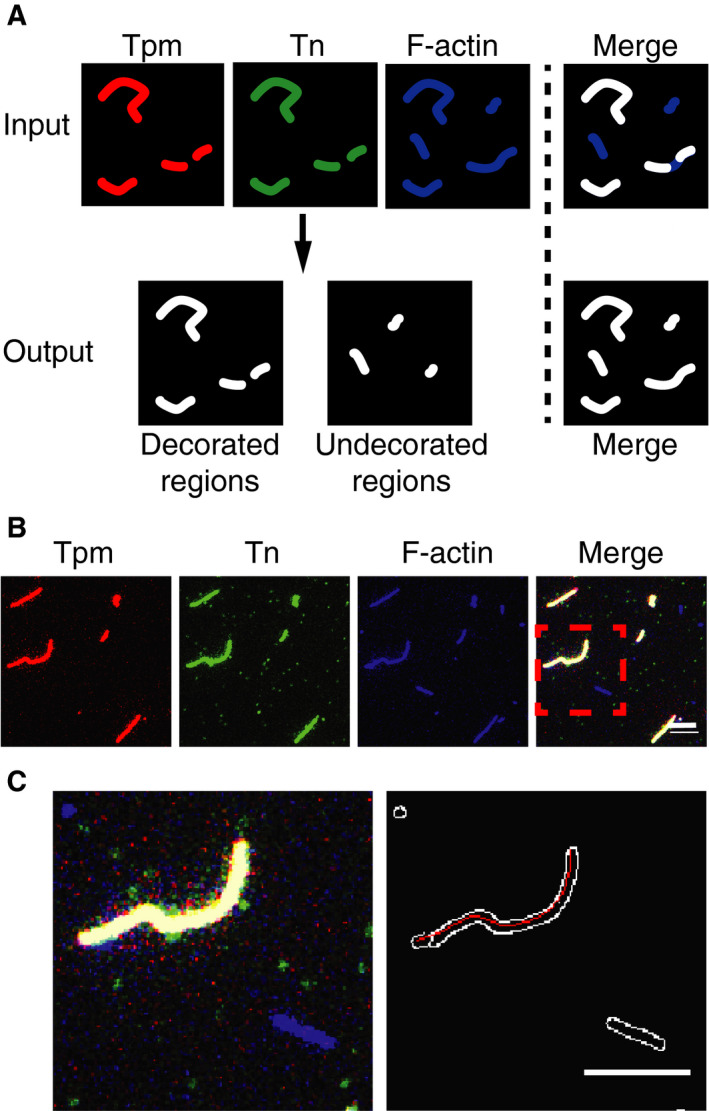
Fluorescence imaging reveals colocalization of the regulatory proteins onto actin filaments. (A) Schematic of the image processing protocol used to distinguish decorated regions depicted by Tpm (red) and Tn (green) from undecorated regions on actin (blue) filaments. (B) Representative epifluorescence images of surface‐deposited thin filaments on aminosilanized coverslips. Reconstitution is at 750 nm, and filaments were diluted to 2 nm for imaging. Images of Tpm (red), Tn (green), F‐actin (blue), and the merged images are shown. Scale bar, 5 μm. (C) Depiction of a high magnification region (left) from (B) shows a fully decorated thin filament (white) and a bare actin filament (blue). The output from the image processing algorithm (right) depicts all actin filament outlines (white) and the identified decorated actin regions as splines (red). Scale bar, 5 μm.

To directly visualize Tn‐Tpm binding to F‐actin, reconstituted filaments were surface‐deposited on aminosilanized coverslips to prevent Brownian motion during image acquisition. Decorated regions were classified as those in which Tn and Tpm colocalize on F‐actin (Fig. 1A; Fig. S2). With stoichiometric reconstitution of thin filaments, it is possible to find fully decorated filaments and undecorated filaments as well (Fig. 1B). Applying the image classification strategy allows to distinguish decorated regions in actin filaments from undecorated (Fig. 1C).

Titrations with increasing Tn‐Tpm concentrations revealed significant aggregation of filaments when Tn‐Tpm exceeded a certain stoichiometric ratio (Fig. S3). Increasing the concentration of free Tn and Tpm also deteriorated the image processing analysis due to fluorescence intensity oversaturation in the field of view. Therefore, Tn‐Tpm binding to F‐actin was studied as a function of the total thin filament concentration (1Tn: 1Tpm: 7 actin monomers). To discard possible effects of phalloidin on filament assembly, thin filaments were reconstituted in the absence of phalloidin AF488 and by covalent labeling of IANBD to Cys 374 in F‐actin (Fig. S4). Neither IANBD labeling nor phalloidin AF488 affects Tn‐Tpm binding. Thus, subsequent experiments used phalloidin AF488 to image actin filaments. In addition, surface deposition of thin filaments on aminosilanized surfaces allows multichannel image acquisition without significant filament motion.

### Binding analysis of Tn‐Tpm to F‐actin by colocalization

**Fig. 2:**
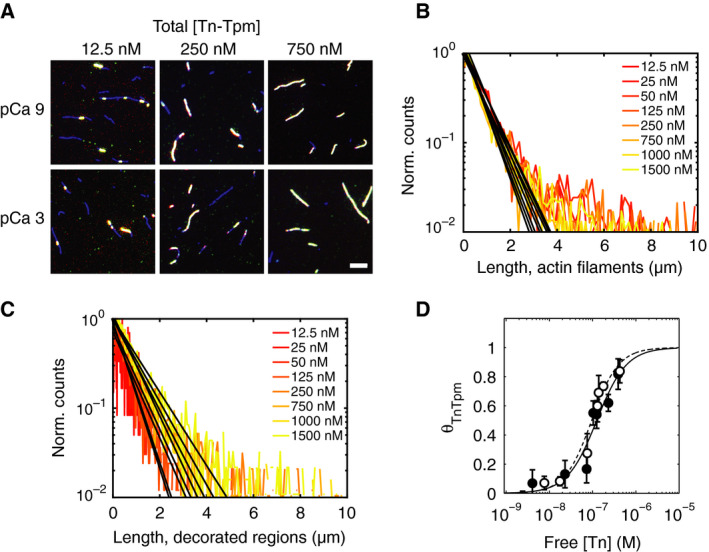
Direct imaging of Tn and Tpm decoration on F‐actin estimates Tn‐Tpm binding affinity. (A) Decorated actin filament reconstituted at varying total protein and Ca^2+^ concentrations shows progressive occupancy of the regulatory proteins Tn and Tpm on actin. Scale bar 5 μm. (B, C) Length distributions show that the decorated regions increase in length with increasing total protein concentration, while the actin filament length remains unchanged. Black lines depict a single‐exponential curve fits. Length distributions at pCa 9 are similar to those at pCa 3. (D) Binding isotherm derived from the length ratios of (B) over (C) as a function of the free Tn concentration at pCa 9 (empty circles) and pCa 3 (filled circles) is shown. Curve fits to the Hill equation at pCa 9 (dashed line) and pCa 3 (continuous line) provide the dissociation constant *K*_d_ and the Hill coefficient *n*_H_ summarized in Table 1. Data points are reported as mean ± SD. *N* = 15 from three independent experiments.

Reconstitution of thin filaments reveals that at low thin filament concentrations (12.5 nm), Tn‐Tpm forms nucleation points on actin filaments (Fig. 2A). Reconstitution at ≥ 500 nm produces thin filament clusters that finally coalesce into fully decorated actin filaments. Filament reconstitution at pCa 3 is indistinguishable from pCa 9 conditions. Binding of Tn or Tpm alone to F‐actin is not visible throughout all the tested conditions. Therefore, thin filament assembly requires the tripartite assembly of Tn, Tpm, and F‐actin.

**Table 1:** Summary of binding curve parameters.

Binding curve	pCa 9		pCa 3	
	*K*_d_ (μm)	*n*_H_	*K*_d_ (μm)	*n*_H_
Saturation (Fig. 2D)	0.68 ± 0.30	1.4 ± 0.1	0.52 ± 0.32	1.3 ± 0.2
Tn‐actin Corr. (Fig. 3B)	0.06 ± 0.03	0.9 ± 0.3	0.04 ± 0.02	0.7 ± 0.3
Tpm‐actin Corr. (Fig. 3C)	0.12 ± 0.05	1.3 ± 0.3	0.07 ± 0.06	1.2 ± 0.1

*n* = 5 analyzed images per data point. Data reported as mean ± asymptotic standard error (α = 0.05).

To clarify the mechanism by which Tn‐Tpm saturate F‐actin binding sites, the length distributions of Tn‐Tpm‐bound regions and F‐actin were studied. Average filament length histograms at each thin filament concentration and free Ca^2+^ concentration show a single‐exponential distribution (Fig. 2B,C; Fig. S6A,B). The single‐exponential F‐actin length distributions seen here are in agreement with previous reports [29]. Decorated regions show increasing length distribution shifts with thin filament concentration, while F‐actin length remains invariant. Length distributions at pCa 3 are indistinguishable from results at pCa 9 (Fig. S5; Fig. S6C,D). Quantification of Tn‐Tpm saturation (Eqn 1) is presented as a function of the free Tn concentration (Fig. 2D). This reveals a *K*_d_ of 0.68 ± 0.32 μm at pCa 9 and 0.52 ± 0.30 μm at pCa 3, respectively. The *K*_d_ values were smaller than in previous reports (~ 2 μm). This is possibly due to the reduced ionic strength used here (125 mm) from that used in previous reports (300 mm) [9]. In addition, Ca^2+^ did not affect *K*_d_ (*P* > 0.05), in agreement with previous reports [9, 30]. The cooperative factor *n_H_* is > 1 at pCa 9 and pCa 3. This suggests that Tn‐Tpm binding is cooperative.

### Binding analysis of Tn‐Tpm to F‐actin by cross‐correlation

**Fig. 3:**
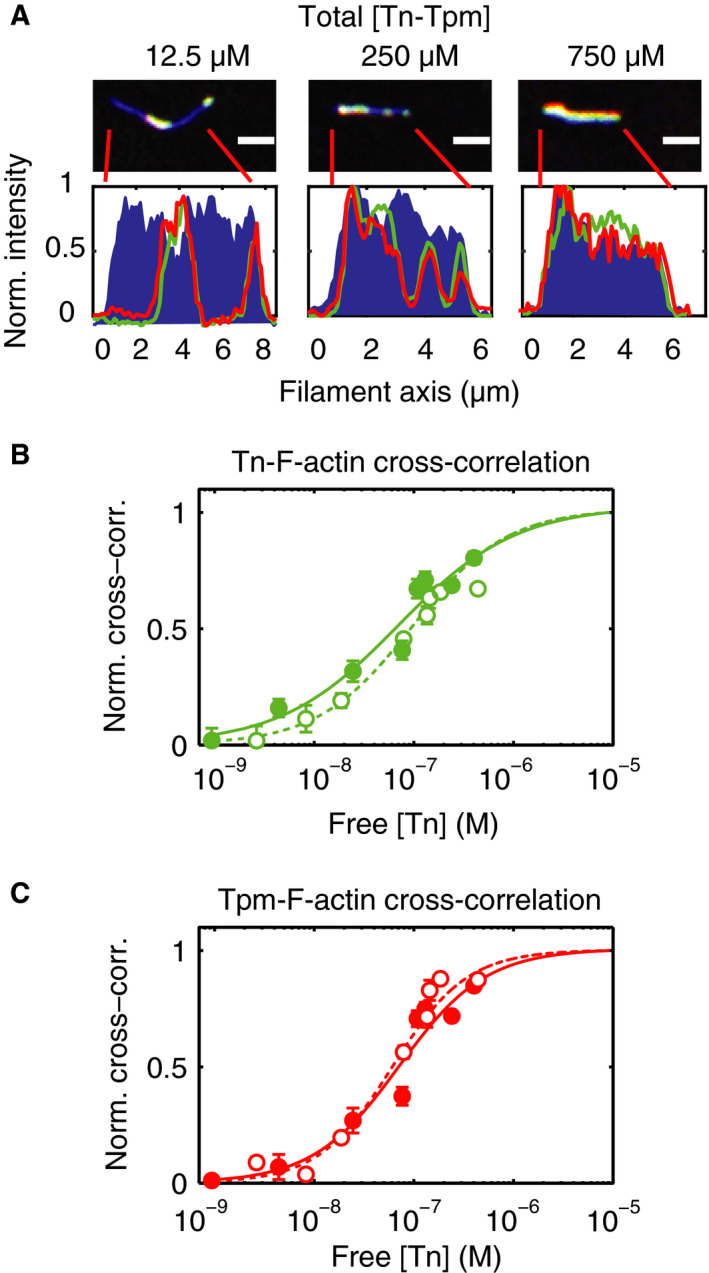
Fluorescence correlation analysis shows that Tn binding affinity is smaller than for Tpm. (A) The fluorescence intensity profile of Tn, Tpm, and F‐actin along the identified F‐actin coordinates is shown at different concentrations of total Tn‐Tpm. Scale bar, 2 μm. The cross‐correlation products between TnC and F‐actin (B) and Tpm and F‐actin (C) at pCa 9 (*open circles*) and pCa 3 (*filled circles*) as a function of the free Tn concentration are shown. Curve fits to the Hill equation for pCa 9 (*dashed line*) and pCa 3 (*continuous line*) provide the dissociation constant *K_d_* and the Hill coefficient *n_H_* (see Table 1). Data points are reported as mean ± SD. N = 1307–2608 and 1205–2834 filaments (from a total of 15 images) for pCa 9 and pCa 3, respectively, derived from three independent experiments.

The intensity profile of Tn and Tpm shows that as the concentration of filaments increases Tn and Tpm binding increases while the vacant regions become less frequent (Fig. 3A). The cross‐correlation between the Tn and F‐actin and Tpm and F‐actin signals is used as a measure of filament saturation (Fig. 3B,C). This reveals that the cooperativity of Tn is lower than for Tpm.

### Modeling of thin filament assembly

**Fig. 4:**
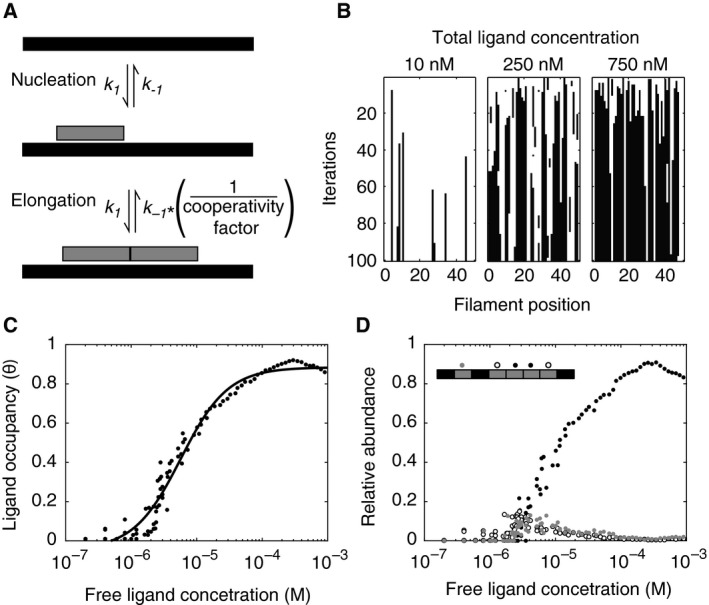
Modeling the mechanism of decoration of thin filaments by Tn‐Tpm recapitulates the in vitro observations. (A) Binding of a single Tn‐Tm unit (nucleation) to a F‐actin lattice with forward and backward stochastic rate constants *k*_1_ and *k*_−1_ precedes the contiguous binding of Tn‐Tm units (elongation). Cooperative effects caused by neighbor‐to‐neighbor interactions between Tn‐Tm units are considered in the simulation by introducing a weighing factor that alters the stochastic dissociation rate constant *k*_−1_ when contiguous ligand interactions exist. (*B*) Simulation traces at 10, 250, and 750 nm of total ligand concentration recapitulate the self‐aggregation process of ligands (black) in the linear lattice (white) seen in the in vitro experiments. (C) Binding isotherm derived from several simulations (black dots) as a function of the free ligand concentration is fit to a Hill equation (black line, *K*_d_ is 0.47 ± 0.24 μm and *n*_H_ is 1.2 ± 0.1; data are reported as mean ± asymptotic standard error). (D) The abundance of each kind of configuration as a function of the free ligand concentration is shown. The three possible configurations correspond to a single ligand bound to the lattice (gray), a ligand flanked by another ligand (open circles), and a ligand flanked by two other ligands (closed circles). The inserted diagram summarizes the described configurations. Steady‐state conditions are reached within 100 iterations. *N* = 100 filaments. The filaments follow an exponential distribution with an average of 40 ligand sites similar to the in vitro actin filaments.

Tn‐Tpm binding to F‐actin was modeled using a Monte Carlo simulation [28]. In the simulation, a Tn‐Tpm unit was considered a single ligand; also, every seven contiguous actin monomers constitute a single binding site in a one‐dimensional lattice [8]. Thus, Tn‐Tpm binds to an isolated binding site with a forward and backward stochastic reaction constants *k*_1_ and *k*_−1_ (Fig. 4A). Two ligands bound contiguously decrease *k*_−1_ proportionally to a cooperativity factor. For simplification, it is assumed that *k*_1_ is not affected by cooperative interactions. Figure 4B illustrates transient reactions reaching equilibrium at different filament concentrations. The lattice saturation for several initial reactant concentrations was measured, and the experimental *K*_d_ was used as a constraint to resolve the respective stochastic reaction constants. It was found that the curve fitted to a Hill equation with positive cooperativity (Fig. 4C). The types of interactions occurring within each ligand bound to the lattice were quantified as a function of the free ligand concentration. These results showed that under low free ligand concentrations (< 10^−7^ m), each ligand was predominantly found isolated from other bound ligands (Fig. 4D). As the free ligand concentration increases, contiguous ligand interactions become more frequent and overtake the number of isolated bound ligands at high concentrations. As shown in Fig. 4D, the relative abundance of each binding state across the titration curve corresponds to 2.7%, 1.5%, and 95.8% for isolated ligands, one‐side, and two‐side interacting ligands. These ratios are derived from the integration of the area under each curve in Fig. 4D.

## Discussion

Here, direct imaging revealed the colocalization of the regulatory proteins Tn and Tpm to actin filaments. This approach estimated the binding affinity of Tn‐Tpm for F‐actin. Fluorescence correlation analysis showed that Tn binding affinity is smaller relative to Tpm. Finally, modeling the mechanism of decoration of thin filaments by Tn‐Tpm recapitulates the in vitro observations.

The introduction of a direct imaging method to determine binding curves has the advantage of minimizing sample artifacts. Moreover, this allowed quantification of Tn and Tpm binding by two approaches. The first method used the colocalization of both Tn and Tpm. The caveat of this approach is that quantification of Tn and Tpm content is limited by the diffraction limit of light (maximum resolution is ~ 250 nm). Alternatively, Tn‐Tpm colocalization was quantified by means of the fluorescence intensity in the decorated regions (Fig. 3A). Because the fluorescence intensity varies between samples, the cross‐correlation product between F‐actin and Tn and F‐actin and Tpm is used as a surrogate method to measure of Tn‐Tpm localization on actin filaments.

Although the *K*_d_ values reported by the two methods are different, the *K*_d_ results are consistent with previous sedimentation studies where *K*_d_ equals 2 μm at 300 mm KCl and Ca^2+^ has no effect on *K*_d_. The ionic strength applied in these studies is lower (125 mm), which explains the increased affinity (*K*_d_ = 0.7–0.5 μm for length‐based and 0.06–0.04 μm for cross‐correlation‐based measurements). The discrepancies between the *K*_d_ values reported here could be due to a higher precision in the determination of bound Tn‐Tpm units at low concentrations of thin filaments by the correlation approach. On the other hand, the correlation approach could have a lower resolution at high ligand occupancies where the fluorescence intensity path adopts a noisier pattern. While the *K*_d_ values found by each method vary, both methods revealed comparable results in relation to the effect of Ca^2+^ on Tn‐Tpm binding.

The fluorescence imaging quantification approach also points to differences between Tn and Tpm binding to F‐actin. The fluorescence correlation data (Fig. 3B,C) suggest that the binding cooperativity for Tn is relatively lower than for Tpm. One possibility is that because the fluorescent probe is bound to TnC, these data are reflecting the difference in the nature of the binding of Tpm to actin when compared to TnC. TnC can be exchanged into skinned fibers without significant dissociation of Tpm and the other Tn subunits [31, 32, 33]. This is consistent with the well‐documented structural information of thin filaments and troponin in which TnI, TnT, and Tpm binding is highly intertwined, while TnC binding relies on comparatively smaller number of contact points with TnI and TnT [3, 34, 35, 36].

Imaging the tripartite constituents of thin filaments did not reveal the presence of Tpm units bound to F‐actin independently of Tn. Single Tpm units bind to F‐actin with a *K*_d_ ≈ 100 μm, while concatenated binding to F‐actin has a *K*_d_ ≈ 0.1 [37, 38]. Under the experimental free Tpm concentrations, it would be possible for Tpm to bind to F‐actin independently of Tn. However, the experimental data do not support this observation (Fig. 2A). One explanation is that Tn and Tpm are at the same concentration. Thus, the probability of having isolated patches of Tpm‐decorated filaments is low.

To better understand the binding mechanism, a stochastic simulation based on an excluded lattice occupancy problem was applied [39, 40] (Fig. 4A). Previous reports have suggested that Tn‐Tpm bind cooperatively to F‐actin [8, 41]. Therefore, Tn‐Tpm binding was modeled as a two‐step reaction process with the second reaction involving cooperative interactions with adjacent Tn‐Tpm units. By using an iterative process, it was possible to obtain a cooperative binding curve with nearest‐neighbor cooperative interactions in a linear lattice. Quantification of the types of interactions present throughout the titration curve revealed that the most common state is that of a ligand bound to adjacent ligands. Single ligands bound to the lattice are more abundant at low concentrations. When this is translated to the in vitro results, the interpretation is that most of the interactions, including those of diffraction‐limited regulatory regions, may represent concatenated Tn‐Tpm ligands that are stable enough to remain bound to F‐actin. This highlights a case in which ligand interactions define the mechanism of saturation of a liner lattice [42, 43].

Although this work highlights the feasibility of employing direct imaging to quantify thin filament assembly, there are additional steps that can improve this technique. One example is to determine more accurately the *K*_d_ by directly quantifying the number of regulatory units found in a decorated region. This could be done by stepwise photobleaching of the fluorescently labeled proteins in a specific area while observing the stepwise decay in fluorescence [44]. Other practical applications of the direct imaging technique may be employed to determine how particular mutations in Tn and Tpm affect their binding to thin filaments [45].

## Conflict of interest

The authors declare no conflict of interest.

## Author contributions

CS and JMR designed research; CS performed experiments; CS performed data analysis and modeling; and CS wrote the manuscript.

## Supporting information


**Fig. S1.** Time‐dependent decay in the localization of Tn‐Tpm on F‐actin. Thin filaments were prepared at 750 nM and diluted to 2 nM. Filaments were surface deposited and imaged at time intervals of 0, 15, 30, 45, 60, 90, 120, and 240 min from the moment that were diluted. Filament reconstitution and determination of the saturation state were conducted as described in Materials and Methods. Data points are reported as mean ± SD. N = 3 images from three independent experiments.
**Fig. S2.** Image processing and analysis workflow. Data sets consist of three images (channels) corresponding to fluorescence emission from Tm (ATTO 655), Tn (TnC‐AF 546), and F‐actin (phalloidin AF488). (A) Binarization and skeletonization in all the images is achieved by applying a Sobel edge filter. Actin skeletons are derived from the F‐actin channel while the decorated actin skeletons are a combination of the Tn and Tm binary images (AND Boolean operation) Skeletonization leads to single pixel‐wide filament layouts that represent the filament coordinates. (B) Tn‐Tpm saturation is the ratio of the number of decorated actin pixels to total actin pixels. (C) The cross correlation products are derived from interrogating the florescence intensity in the all three channels using the actin skeleton coordinates. Once the Tn, Tpm and F‐actin signals in a filament are obtained, the cross‐correlation product of the Tn‐to‐F‐actin and Tn‐to‐F‐actin are calculated. The filament analysis algorithm can be found in an online repository (https://doi.org/10.5281/zenodo.844151).
**Fig. S3.** Saturation of F‐actin filaments with Tn‐Tm. Thin filaments were reconstituted with Tn (TnC 89C AF546), Tpm (190C ATTO 655), and F‐actin (phalloidin AF488) as described in Materials and Methods. (*A*) Regulated actin filaments were incubated at different ratios of Tn‐Tm relative to F‐actin (0.5 µM). As the Tn‐Tm concentration increases, the filament aggregation becomes more frequent, and the number of filaments per field of view decreases. (*B*) Representative filament bundle at 5 µM Tn‐Tm. Scale bar, 5 µm.
**Fig. S4.** Stability of thin filaments. (*upper panel*) Thin filaments were reconstituted with Tn (TnC 89C AF546), Tpm (190C ATTO 655), and F‐actin (374C IANBD); and (*lower panel*) Tn (TnC 89C AF546), Tm (190C ATTO 655) and F‐actin. Scale bar, 5 µm. IANBD was imaged with the same filter combination for AF488 while the image acquisition settings of Tn and Tpm were unchanged.
**Fig. S5.** Length distributions of total F‐actin and decorated actin regions at pCa3. Length distributions at pCa 3 (A) show that the decorated regions increase in length with increasing total protein concentration while the actin filament length remains unchanged similarly to pCa 9 conditions (B). Black line depicts a single‐exponential curve fit. Related to Fig. 2. N = 1205‐2834 filaments from three independent experiments. Each experiment consisted of five image captures distributed uniformly throughout the cover slip.
**Fig. S6.** Length distributions of total F‐actin and decorated actin regions. Graphs represent length distributions of (A) F‐actin at pCa 9, (B) regulated regions at pCa 9, (C) F‐actin at pCa 3, and (D) regulated actin regions at pCa3. Plots correspond to non‐logarithmic representations of Fig. 2 C,D and Fig. S5 A,B.

## Data Availability

The image processing algorithm and the simulation algorithm used in Fig. 4 are found in a public repository (https://doi.org/10.5281/zenodo.844151 and https://doi.org/10.5281/zenodo.844153, respectively).
