# Supplementary material for: Cardiac troponin and tropomyosin bind to F‐actin cooperatively, as revealed by fluorescence microscopy
Source: FEBS Open Bio. 2020 Jun 18;10(7):1362–72. doi: 10.1002/2211-5463.12876 (PMC7327902; doi:10.1002/2211-5463.12876)
Supplement: Supplementary file 1 — Fig. S1. Time‐dependent decay in the localization of Tn‐Tpm on F‐actin. Thin filaments were prepared at 750 nM and diluted to 2 nM. Filaments were surface deposited and imaged at time intervals of 0, 15, 30, 45, 60, 90, 120, and 240 min from the moment that were diluted. Filament reconstitution and determination of the saturation state were conducted as described in Materials and Methods. Data points are reported as mean ± SD. N = 3 images from three independent experiments. Fig. S2. Image processing and analysis workflow. Data sets consist of three images (channels) corresponding to fluorescence emission from Tm (ATTO 655), Tn (TnC‐AF 546), and F‐actin (phalloidin AF488). (A) Binarization and skeletonization in all the images is achieved by applying a Sobel edge filter. Actin skeletons are derived from the F‐actin channel while the decorated actin skeletons are a combination of the Tn and Tm binary images (AND Boolean operation) Skeletonization leads to single pixel‐wide filament layouts that represent the filament coordinates. (B) Tn‐Tpm saturation is the ratio of the number of decorated actin pixels to total actin pixels. (C) The cross correlation products are derived from interrogating the florescence intensity in the all three channels using the actin skeleton coordinates. Once the Tn, Tpm and F‐actin signals in a filament are obtained, the cross‐correlation product of the Tn‐to‐F‐actin and Tn‐to‐F‐actin are calculated. The filament analysis algorithm can be found in an online repository (https://doi.org/10.5281/zenodo.844151). Fig. S3. Saturation of F‐actin filaments with Tn‐Tm. Thin filaments were reconstituted with Tn (TnC 89C AF546), Tpm (190C ATTO 655), and F‐actin (phalloidin AF488) as described in Materials and Methods. (A) Regulated actin filaments were incubated at different ratios of Tn‐Tm relative to F‐actin (0.5 µM). As the Tn‐Tm concentration increases, the filament aggregation becomes more frequent, and the number of filaments per field of view de [file FEB4-10-1362-s001.pdf]

Supporting Material

Cardiac troponin and tropomyosin bind to F-actin cooperatively, as revealed by fluorescence microscopy

Christopher Solís and John M. Robinson

Department of Chemistry and Biochemistry, South Dakota State University, Brookings, South Dakota 57007

Supplementary Figures

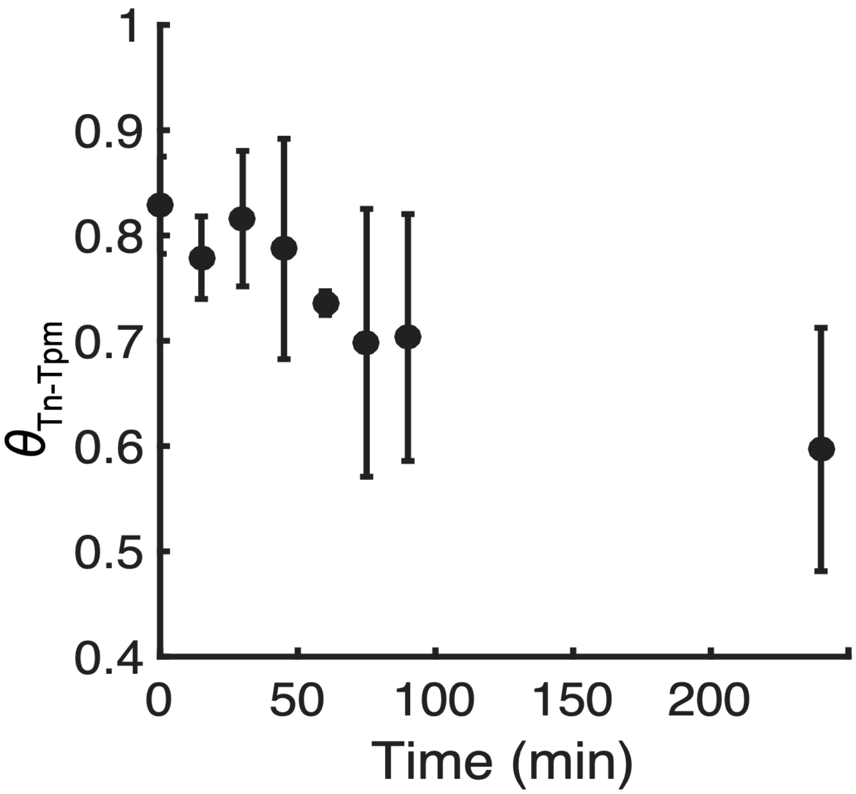

Fig. S1. Time-dependent decay in the localization of Tn-Tpm on F-actin. Thin filaments were prepared at 750 nM and diluted to 2 nM. Filaments were surface deposited and imaged at time intervals of 0, 15, 30, 45, 60, 90, 120, and 240 min from the moment that were diluted. Filament reconstitution and determination of the saturation state were conducted as described in Materials and Methods. Data points are reported as mean ± SD. N = 3 images from three independent experiments.

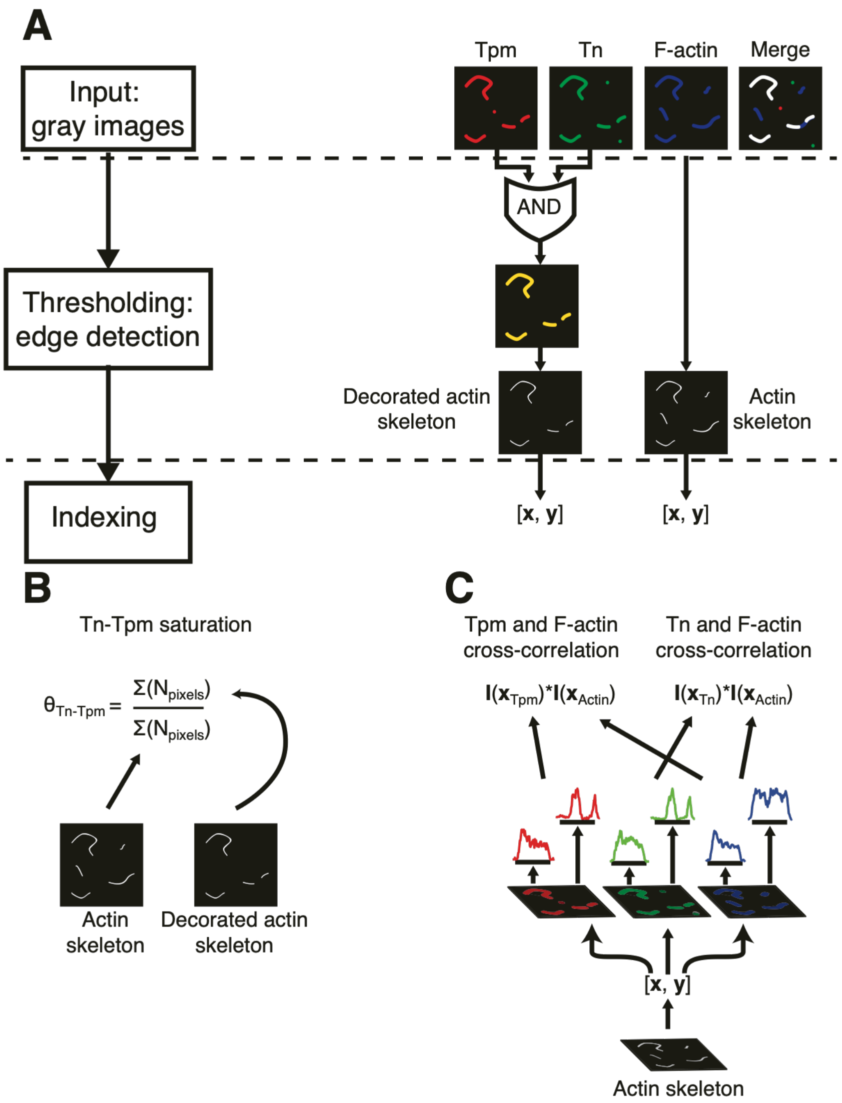

Fig. S2. Image processing and analysis workflow. Data sets consist of three images (channels) corresponding to fluorescence emission from Tm (ATTO 655), Tn (TnC-AF 546), and F-actin (phalloidin AF488). (A) Binarization and skeletonization in all the images is achieved by applying a Sobel edge filter. Actin skeletons are derived from the F-actin channel while the decorated actin skeletons are a combination of the Tn and Tm binary images (AND Boolean operation) Skeletonization leads to single pixel-wide filament layouts that represent the filament coordinates. (B) Tn-Tpm saturation is the ratio of the number of decorated actin pixels to total actin pixels. (C) The cross correlation products are derived from interrogating the florescence intensity in the all three channels using the actin skeleton coordinates. Once the Tn, Tpm and F-actin signals in a filament are obtained, the cross-correlation product of the Tn-to-F-actin and Tn-to-F-actin are calculated. The filament analysis algorithm can be found in an online repository (https://doi.org/10.5281/zenodo.844151).

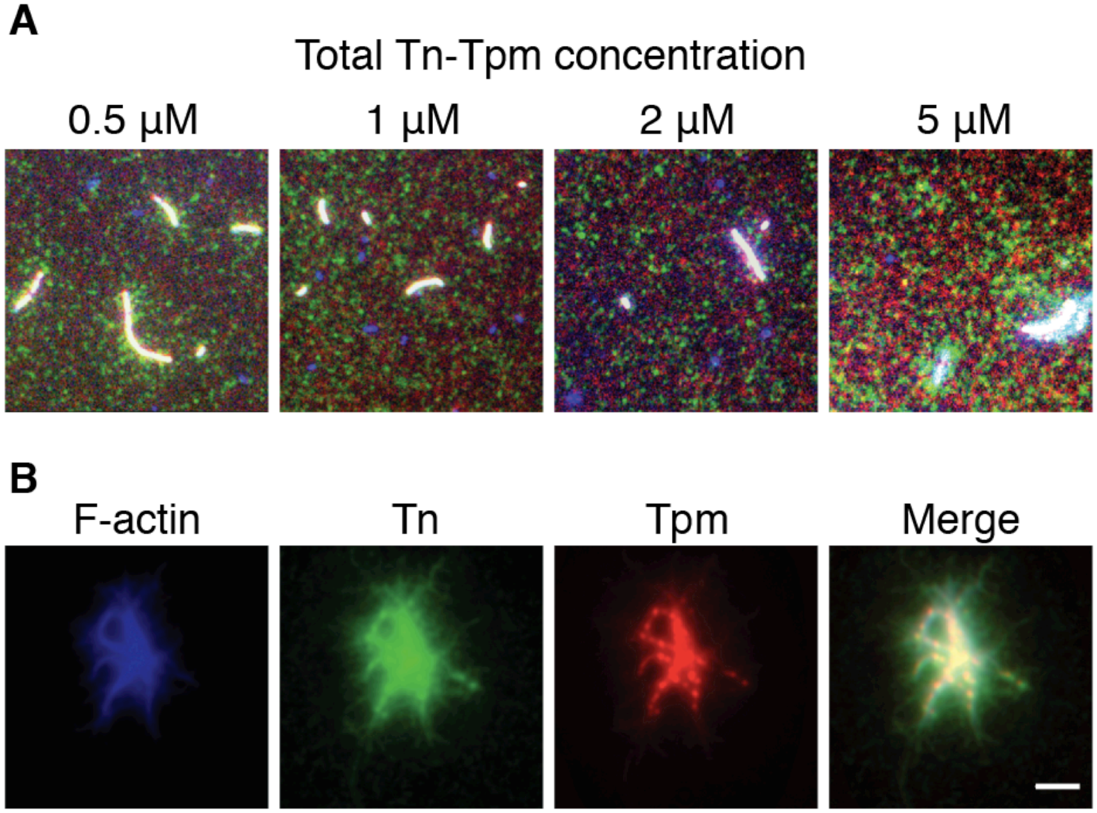

Fig. S3. Saturation of F-actin filaments with Tn-Tm. Thin filaments were reconstituted with Tn (TnC 89C AF546), Tpm (190C ATTO 655), and F-actin (phalloidin AF488) as described in Materials and Methods. (*A*) Regulated actin filaments were incubated at different ratios of Tn-Tm relative to F-actin (0.5 µM). As the Tn-Tm concentration increases, the filament aggregation becomes more frequent, and the number of filaments per field of view decreases. (*B*) Representative filament bundle at 5 µM Tn-Tm. Scale bar, 5 µm.

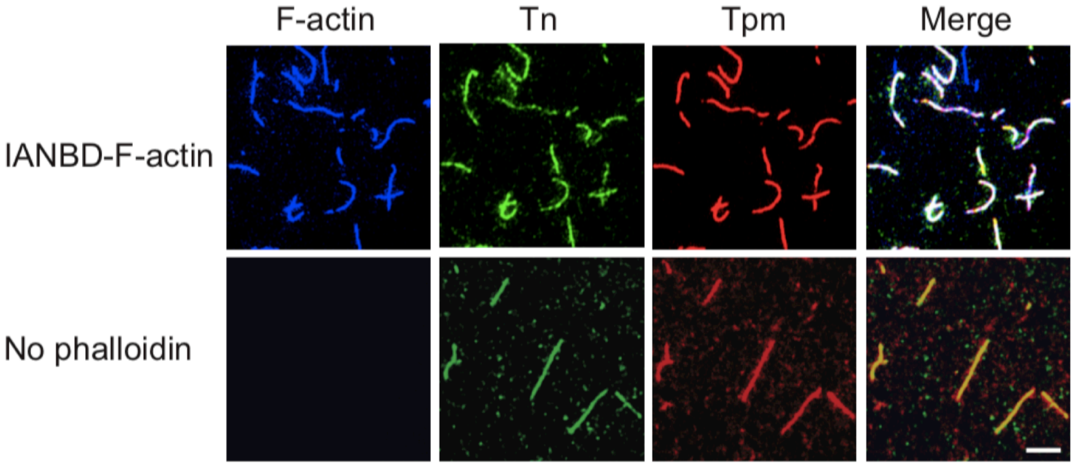

Fig. S4. Stability of thin filaments. (*upper panel*) Thin filaments were reconstituted with Tn (TnC 89C AF546), Tpm (190C ATTO 655), and F-actin (374C IANBD); and (*lower panel*) Tn (TnC 89C AF546), Tm (190C ATTO 655) and F-actin. Scale bar, 5 µm. IANBD was imaged with the same filter combination for AF488 while the image acquisition settings of Tn and Tpm were unchanged.

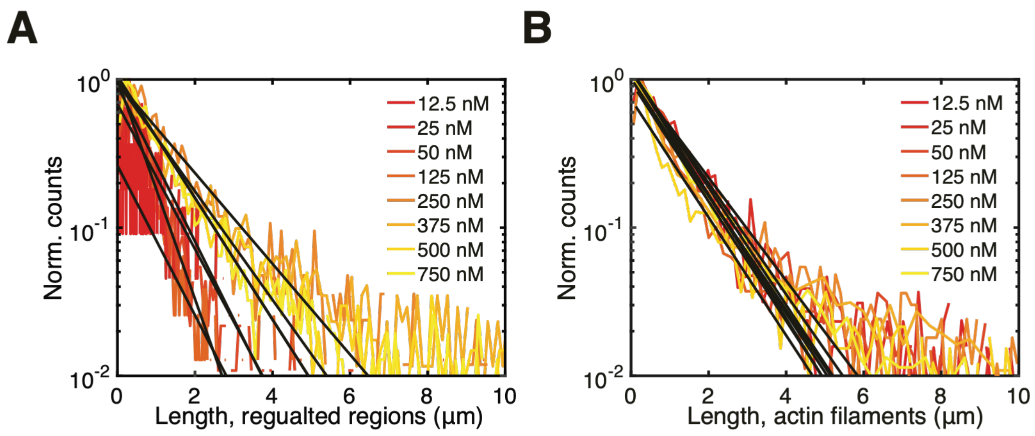

Fig. S5. Length distributions of total F-actin and decorated actin regions at pCa3. Length distributions at pCa 3 (A) show that the decorated regions increase in length with increasing total protein concentration while the actin filament length remains unchanged similarly to pCa 9 conditions (B). Black line depicts a single exponential curve fit. Related to Fig. 2. N = 1205-2834 filaments from three independent experiments. Each experiment consisted of five image captures distributed uniformly throughout the cover slip.

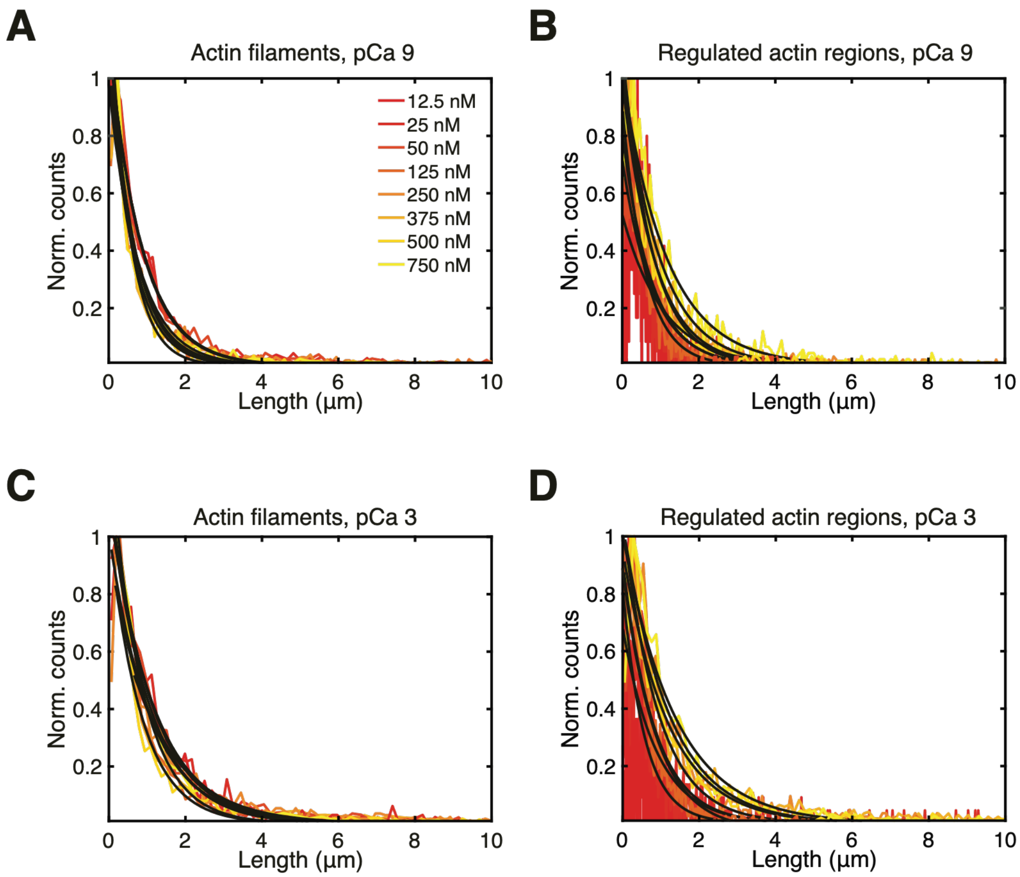

Fig. S6. Length distributions of total F-actin and decorated actin regions. Graphs represent length distributions of (A) F-actin at pCa 9, (B) regulated regions at pCa 9, (C) F-actin at pCa 3, and (D) regulated actin regions at pCa3. Plots correspond to non-logarithmic representations of Fig. 2 C,D and Fig. S5 A,B.

Stochastic Simulation Algorithms

The simulation algorithm used in Fig. 4 is found in a public repository (https://doi.org/10.5281/zenodo.844153).
